# Axillary lymph nodes metastasis in a patient with recurrent papillary thyroid cancer: a case report

**DOI:** 10.1186/s13256-015-0668-7

**Published:** 2015-08-26

**Authors:** Mohamed T. Hafez, Basel Refky, Khaled Abd Elwahab, Mohammad Arafa, Islam Abdou, Waleed Elnahas

**Affiliations:** 1 Surgical Oncology Unit, Oncology Center – Mansoura University, Mansoura, Egypt; 2 Pathology Department, Mansoura Faculty of Medicine, Mansoura, Egypt

## Abstract

**Introduction:**

Thyroid cancer is the most common endocrine malignancy; the most common type of thyroid cancer is papillary thyroid cancer which accounts for approximately 90% of all thyroid cancers. Previously defined prognostic factors of papillary thyroid cancer include age, gender, tumor size, extrathyroidal extension, and distant metastasis. Cervical lymph node metastases are very common in patients with papillary thyroid cancer. Although papillary thyroid cancer has an excellent prognosis, lymphatic spread is associated with an increased risk of locoregional recurrence.

Axillary metastasis is not a common finding in the classic type of papillary carcinoma; hence, a limited number of case reports have described the exceptional and rare metastatic spread of papillary thyroid carcinomas to the axilla.

**Case presentation:**

We report a case of metastatic axillary lymphadenopathy in a 61-year-old Egyptian man with a recurrent papillary thyroid cancer. He had a history of total thyroidectomy with right radical neck dissection 18 months ago. He presented to our cancer clinic at the Oncology Centre –Mansoura University with recurrent mass at the right lower parotid region, left cervical lymphadenopathy and left axillary lymphadenopathy.

Removal of the recurrent right intraparotid mass, left comprehensive neck dissection and left axillary dissection were performed and the postoperative pathology report showed infiltration of the cervical and axillary lymph nodes by metastatic papillary thyroid cancer.

**Conclusions:**

Axillary lymph node enlargement in a patient with papillary thyroid cancer should be considered metastatic from thyroid until proved otherwise. Careful thorough examination of patients with recurrent thyroid cancer is essential to address any unusual metastasis.

## Introduction

Papillary thyroid cancer (PTC) is the most common form of differentiated thyroid cancer, comprising approximately 90% of the new cases of thyroid cancer in the USA [1]. Surgery is the definitive management of PTC. Cervical lymph node metastases (LNM) are common in PTC and are associated with a significant probability for locoregional recurrence of the disease, even in low-risk patients. These considerations generated a strong interest in a more comprehensive preoperative evaluation of the neck and renewed the controversy about the role and the extent of lymphadenectomy at the time of thyroidectomy [2]. Careful preoperative clinical examination and neck imaging are very critical to not miss any LNM.

Regional LNM are extremely common (up to 50%) at initial presentation of PTC. This feature does not apparently adversely affect long-term prognosis especially in patients under 45 years of age at diagnosis [3]. The usual pattern of cervical LNM of PTC is through central and lateral neck compartments as well as superior mediastinal compartment. Axillary LNM as part of the disease spectrum of thyroid carcinoma is rare, with only isolated case reports [4–10].

## Case presentation

We report a case of axillary lymphadenopathy in a 61-year-old Egyptian man who presented to our cancer clinic at Mansoura Oncology Centre with a recurrent PTC. He had a history of total thyroidectomy and right radical neck dissection 18 months ago, which were followed by two doses of radioactive iodine at intervals of 6 and 12 months.

On clinical examination, there was a recurrent mass at the right lower parotid region, left cervical and axillary lymphadenopathy.

**Fig. 1:**
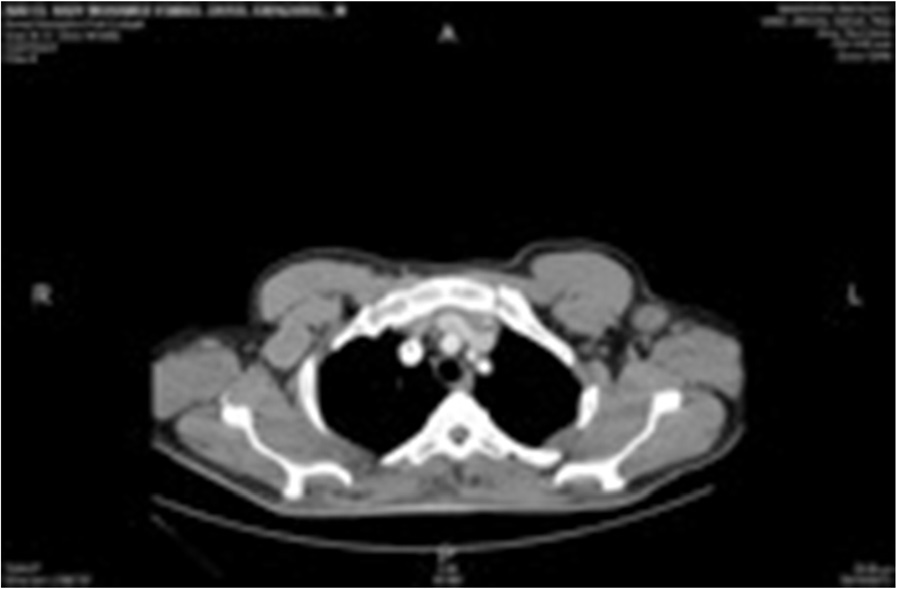
Computed tomography showing significant axillary lymph node

Imaging was performed in the form of a computed tomography (CT) scan of his neck, chest and abdomen which showed: 1) multiple bilateral cervical lymphadenopathies of which the largest node was 2.5cm in diameter at level III, 2) right intraparotid lesion 2.5×3.5cm in diameter, and 3) left axillary lymph nodes of which the largest node was 3cm in diameter (Fig. 1).

Fine-needle aspiration from his left cervical lymph nodes as well as from his left axillary lymph node revealed metastatic PTC.

**Fig. 2:**
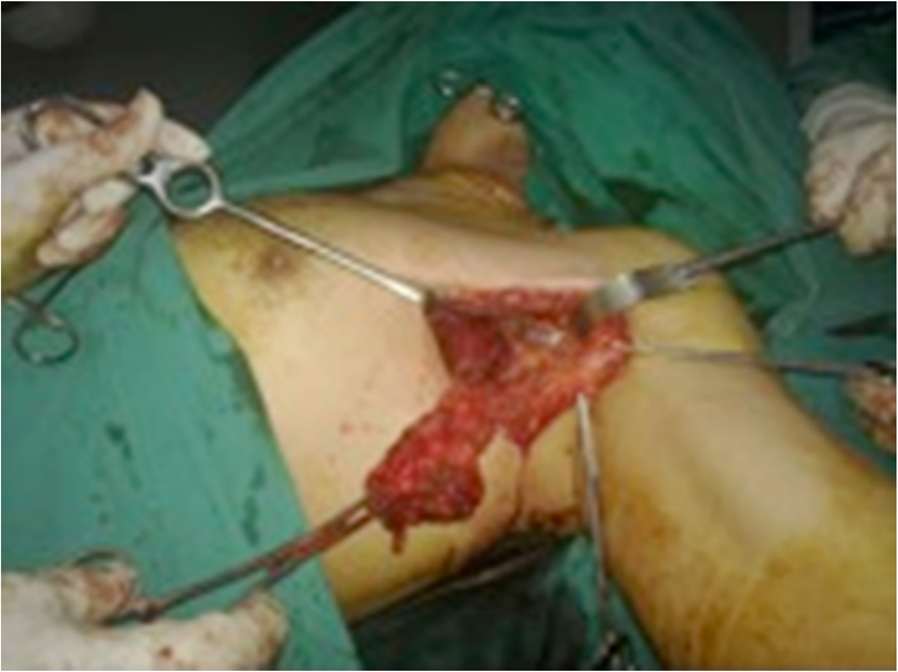
Intraoperative picture of the patient undergoing axillary block dissection after closure of neck wound

He was managed by removal of the recurrent right intraparotid mass, left comprehensive neck dissection and left axillary dissection (Fig. 2).

**Fig. 3:**
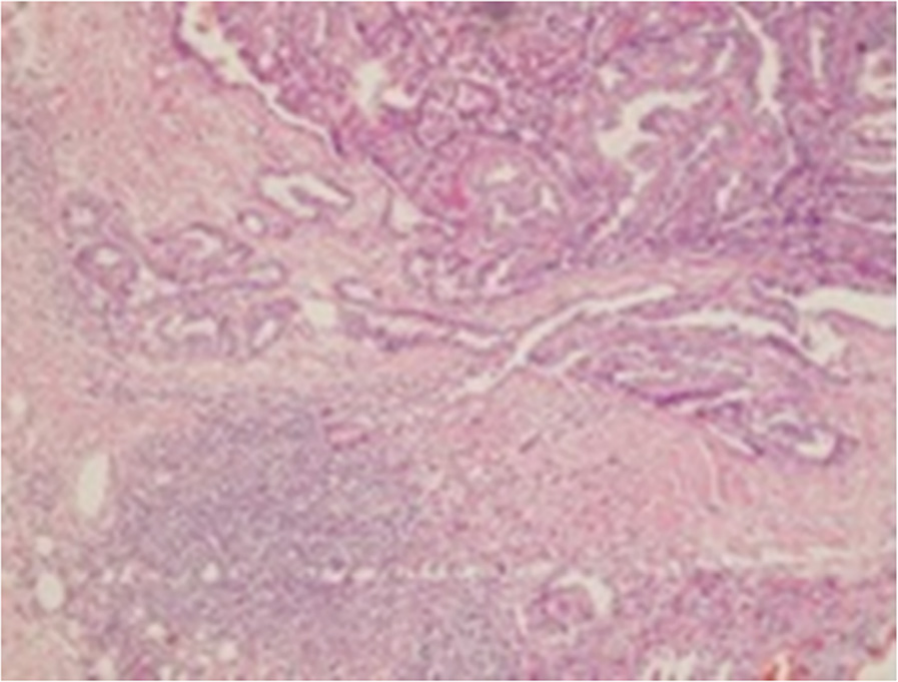
Histopathology of axillary lymph node showing metastatic papillary cancer

Postoperative pathology revealed recurrent papillary cancer at the right intraparotid mass, infiltration of 10 lymph nodes out of 16 lymph nodes of the left neck dissection and infiltration of one lymph node out of 11 lymph nodes in axilla by PTC (Fig. 3).

**Fig. 4:**
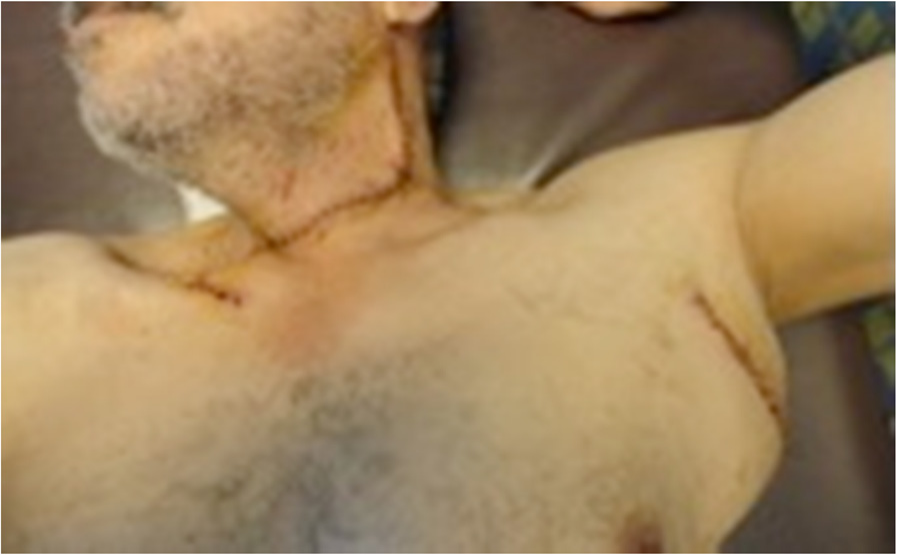
Postoperative picture of the patient 10 days after surgery

He had an excellent recovery without complications (Fig. 4).

## Discussion

PTC and the follicular variant of PTC have a propensity for cervical lymphatic spread that occurs in up to 50% of patients on standard review of surgical pathologic specimens and in 90% of those examined for micrometastases [2, 11].

Regional lymph nodes are located in three compartments: central, lateral and mediastinal [12].

Axillary LNM from thyroid carcinoma is exceedingly rare. A search of the English language literature found only nine cases including our case. The last case report was published in 2011 [4]. The mean age was 55 years, with a patient aged as young as 21 years [6]. Three patients presented with concurrent axillary LNM, and five presented with axillary LNM as a part of the recurrent disease process [5, 8–10].

In cases with recurrent disease process, the period from initial diagnosis to the development of axillary LNM ranged from 5 to 41 years; this was not the same in our case, which was only 18 months. The primary cancer was PTC in eight patients and poorly differentiated adenocarcinoma in one patient. There has been no report of axillary LNM from follicular thyroid carcinoma.

In all final pathology reports that specified tumor differentiation, all patients had poorly differentiated components suggesting that axillary LNM may be associated with poorly differentiated thyroid carcinoma. Seven patients out of the nine had synchronous or metachronous distant metastasis. Axillary LNM may thus be an indicator of systemic disease and a poor prognosis.

**Fig. 5:**
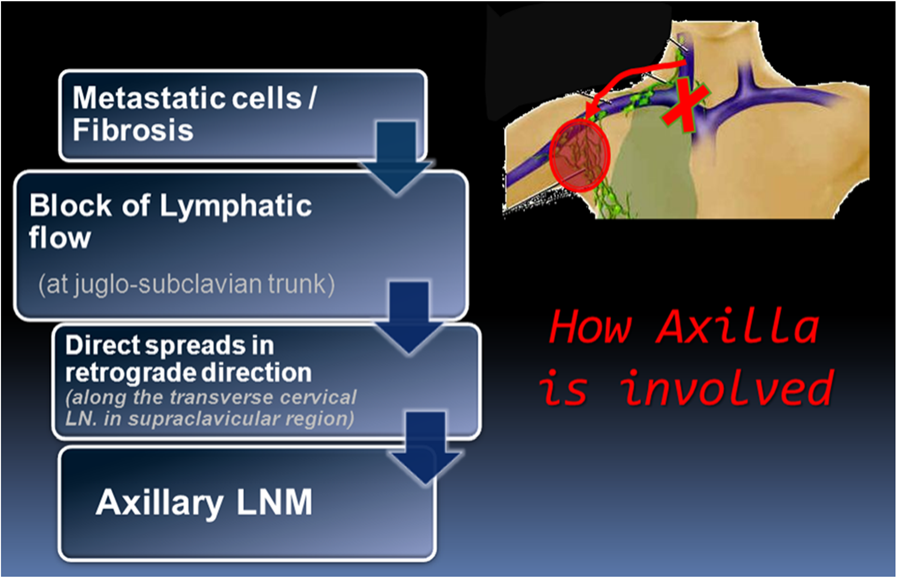
How an axilla is involved. *LN* lymph node, *LNM* lymph node metastases

Rouviere reported that there is a communication between the cervical and axillary lymphatics; however, the physiologic flow is centripetal to the jugulo-subclavian junction [13]. Consequently, mediastinal LNM sometimes occurs in PTC but still axillary LNM is rare. However, malignant tumors can alter and partially block lymphatic pathways, potentially resulting in axillary LNM. When sentinel nodes around the lymphatic terminus in the jugulo-subclavian confluence are involved with carcinoma and their lymphatic flow is blocked, disease spreads in a retrograde direction along the transverse cervical lymph nodes in the supraclavicular region. These retrograde pathways of lymphatic drainage can ultimately culminate in axillary LNM (Fig. 5). In our case we have been able to identify a large lymph node at the left jugulo-subclavian junction.

In summary, we reported a case of axillary LNM from recurrent PTC. Both the recurrent tumor and lymphadenopathy from the neck to axilla were resected with an aim to cure the patient. Axillary LNM is rare and appears to indicate a poor prognosis.

## Conclusions

Axillary lymph node enlargement in a patient with PTC should be considered metastatic from thyroid until proved otherwise.

Careful thorough examination of patients with recurrent thyroid cancer is essential to address any unusual metastasis.

## Consent

Written informed consent was obtained from the patient for publication of this case report and any accompanying images. A copy of the written consent is available for review by the Editor-in-Chief of this journal.

## Abbreviations

LNM: Lymph node metastases
PTC: Papillary thyroid cancer
